# Recurrence After Primary Lumbar Discectomy: Rate, Reoperation, and Risk Factors in A Single‐Center Cohort Over A 10‐Year Study Period

**DOI:** 10.1111/os.70460

**Published:** 2026-11-09

**Authors:** Serhat Yıldızhan, Usame Rakip, İhsan Canbek, Mehmet Gazi Boyacı, Serhat Korkmaz, Adem Aslan

**Affiliations:** ^1^ Department of Neurosurgery Afyonkarahisar Health Sciences University Afyonkarahisar Turkey

**Keywords:** comorbidity, diabetes mellitus, discectomy, hypertension, lumbar disc herniation, recurrence, reoperation, risk factors

## Abstract

**Objective:**

Recurrent lumbar disc herniation after primary discectomy is a clinically important complication whose temporal pattern, revision surgical profile, and associated comorbidities remain incompletely characterized in long‐term cohorts. We aimed to define the rate and timing of reoperation‐confirmed recurrence, characterize the revision profile, and examine associated risk factors.

**Methods:**

This retrospective cohort study included 432 consecutive patients who underwent primary lumbar discectomy between January 2015 and December 2024, with a median follow‐up of 85 months (interquartile range 50–103). Recurrence was defined as reoperation‐confirmed, same‐level ipsilateral reherniation after an initial pain‐free interval. Follow‐up status and external reoperations were verified through the national electronic health record system up to the study end date (6 March 2026), with no loss to follow‐up. Associations were examined using multivariable logistic regression (with Firth penalization) and Cox proportional hazards modeling.

**Results:**

Reoperation‐confirmed recurrence occurred in 28 patients (6.5%); this represents the reoperation‐confirmed recurrence rate rather than the overall incidence of recurrent herniation, since conservatively managed recurrences were not counted. Recurrence was predominantly late, with 68% of events beyond the first postoperative year (median 18.5 months). At reoperation, 21 patients (75%) required instrumented posterolateral fusion for objective segmental instability, with a mean segmental translation of 4.2 ± 1.1 mm and a mean angulation of 12.2° ± 2.1° on dynamic flexion–extension radiographs, both exceeding the predefined instability thresholds. The remaining 7 patients (25%) underwent revision discectomy alone. No second recurrence occurred over a median post‐revision follow‐up of 61.5 months. In the primary logistic model, hypertension was associated with recurrence (adjusted odds ratio [OR] 3.42, 95% confidence interval [CI] 1.04–11.21; *p* = 0.043), whereas the association of diabetes mellitus was of similar magnitude but did not reach statistical significance (OR 3.17, 95% CI 0.97–10.35; *p* = 0.056). Diabetes mellitus reached significance only in the secondary Firth penalized and time‐to‐event analyses. Active smoking was not associated with recurrence (OR 0.64; *p* = 0.321). Given the limited number of events (*n* = 28), these findings should be regarded as hypothesis‐generating rather than confirmatory; in particular, the study was not powered to overturn existing evidence linking smoking with recurrence, and the 10‐year survival estimate is exploratory given few patients at risk beyond 5 years.

**Conclusion:**

In this 10‐year single‐center cohort, reoperation‐confirmed recurrence occurred in 6.5% of patients and was predominantly late (median 18.5 months), indicating that postoperative surveillance should extend beyond the first year. Three quarters of revisions required instrumented fusion for objective instability. No second recurrence was observed. Hypertension was associated with recurrence in the primary analysis, whereas the association of diabetes mellitus did not reach significance in the primary model and was supported only by secondary analyses. Both comorbidities should be investigated further in adequately powered multicenter studies.

## Graphical abstract

In 432 patients from a single center (median follow‐up 85 months), reoperation‐confirmed recurrence after lumbar discectomy occurred in 6.5% and was predominantly late (median 18.5 months). Hypertension was associated with recurrence; diabetes reached significance only in secondary analyses; smoking was not associated. No second recurrence occurred after revision.

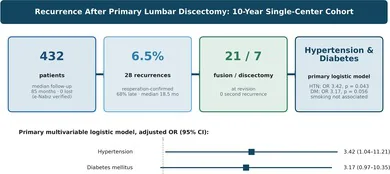

**Abbreviations**
AIC: Akaike information criterion
BMI: body mass index
CI: confidence interval
CSF: cerebrospinal fluid
DM: diabetes mellitus
EPV: events per variable
HR: hazard ratio
HTN: hypertension
IQR: interquartile range
KM: Kaplan–Meier
LDH: lumbar disc herniation
MRI: magnetic resonance imaging
OR: odds ratio
SD: standard deviation
STROBE: Strengthening the Reporting of Observational Studies in Epidemiology
VIF: variance inflation factor
WFNS: World Federation of Neurosurgical Societies

## 1 Introduction

Lumbar disc herniation (LDH) with radiculopathy is one of the most frequent indications for spinal surgery worldwide, imposing a substantial burden on both healthcare systems and individual patients. The annual incidence of surgically treated LDH ranges from 0.3–2.7 per 1000 individuals across different healthcare settings, and global estimates suggest that tens of millions of patients undergo lumbar spine surgery each year [1, 2, 3]. Although conservative management successfully resolves symptoms in the majority of patients, approximately 9%–11% develop progressive neurological deficits, intractable radicular pain, or cauda equina syndrome that require surgical decompression [4, 5, 6]. In such cases, lumbar discectomy is the cornerstone of surgical management, offering excellent short‐term outcomes with success rates exceeding 80% in appropriately selected patients [7, 8, 9, 10].

However, despite favorable initial outcomes [5], lumbar discectomy does not preclude subsequent lumbar pathology. According to long‐term follow‐up data from large national cohorts, a considerable proportion of patients require additional lumbar surgery, with cumulative reoperation rates of approximately 13%–14% at 5 years [11, 12]. Among reasons for reoperation, recurrent disc herniation at the index level is the most common finding, and its management frequently necessitates more invasive strategies, including instrumented fusion, with correspondingly greater morbidity and cost than the primary procedure [13]. Reported recurrence rates after primary discectomy vary considerably, ranging from 5%–11%, reflecting heterogeneity in patient selection, surgical technique, recurrence definition, and follow‐up duration [13, 14, 15]. Although reliable identification of high‐risk patients remains clinically relevant, two further aspects of recurrent disease, namely its temporal pattern and the surgical profile at revision, bear directly on postoperative surveillance and operative planning, yet remain less well characterized in long‐term cohorts.

Considerable investigative effort has been directed toward identifying patient‐related risk factors for recurrent LDH. In earlier meta‐analyses, cigarette smoking was the most frequently identified predictor, with pooled risk estimates indicating a near‐doubling of recurrence risk [16]. Beyond smoking, diabetes mellitus has been associated with recurrence through mechanisms related to impaired disc nutrition and accelerated degeneration [17]. The contribution of hypertension has also been proposed, as systemic hypertension may induce microvascular insufficiency in avascular disc tissue, compromising annular healing capacity following discectomy [18]. However, existing studies are limited by differences in study design, short follow‐up periods, small numbers of recurrence events, and inconsistent adjustment for confounding variables, leaving the relative importance and temporal behavior of these risk factors incompletely characterized [18, 19]. Of comparable clinical importance, two aspects of recurrent disease remain insufficiently defined in long‐term series: the temporal distribution of recurrences, specifically whether most events occur early or late in the postoperative course, which directly governs the design of follow‐up programs, and the surgical profile at revision, particularly the proportion of cases requiring instrumented fusion rather than simple re‐discectomy. Long‐term data characterizing these two parameters, however, remain scarce.

Given these unresolved questions, a longitudinal analysis integrating the recurrence rate, temporal pattern, reoperation strategy, and multivariate risk factor assessment within a single cohort is needed. We therefore conducted a retrospective cohort study of 432 patients who underwent primary lumbar discectomy over a 10‐year period at a single tertiary academic neurosurgical center. The specific objectives were: (1) to determine the rate and temporal distribution of recurrence requiring reoperation, with particular attention to the proportion of late (beyond one year) events; (2) to characterize the surgical management profile at revision, including the proportion requiring instrumented fusion and the incidence of second recurrence; and (3) to examine the association of candidate risk factors with recurrence using complementary logistic regression and time‐to‐event analyses, while explicitly acknowledging the statistical constraints imposed by the limited number of events.

## 2 Methods

### 2.1 Study Design and Patient Selection

This retrospective cohort study was conducted at the Department of Neurosurgery of a single tertiary academic center. The study was approved by the institutional Clinical Research Ethics Committee prior to data collection (meeting No. 2026/3, decision No. 208, dated 6 March 2026) and was conducted in full accordance with the Declaration of Helsinki. This report adheres to the Strengthening the Reporting of Observational Studies in Epidemiology (STROBE) guidelines for cohort studies.

The study population comprised consecutive patients who underwent primary lumbar discectomy between January 2015 and December 2024. Patients were included if they met all of the following criteria: (1) first‐time lumbar discectomy for extruded or sequestrated disc herniation confirmed by preoperative magnetic resonance imaging (MRI); (2) clinical presentation consistent with radicular pain or neurological deficit attributable to the documented herniation; and (3) a minimum postoperative follow‐up of 12 months. The following were excluded: disc protrusion without a clear surgical indication; acute cauda equina syndrome requiring emergency decompression; spinal neoplasm or infection at any level; traumatic disc herniation; prior lumbar spinal surgery at any level; and concomitant spinal stenosis or spondylolisthesis requiring additional instrumentation at the index procedure.

### 2.2 Surgical Technique

All index procedures were performed by the same neurosurgical team using a standardized posterior microsurgical approach [8] under general anesthesia. After intraoperative fluoroscopic localization of the target level, a unilateral interlaminar approach was employed through a midline incision. Hemilaminotomy and partial flavectomy were performed to expose the lateral dural sac and affected nerve root. The dural sac was gently retracted medially, and the herniated disc fragment was removed in its entirety. Limited exploration of the disc space was undertaken to retrieve any loose or migrated fragments; aggressive intradiscal curettage was avoided to minimize segmental destabilization. Annular defects were not primarily repaired, and no annular closure device or biological barrier material was used in any patient. Perioperative variables, including intraoperative complications and length of hospital stay, were extracted from institutional records. For patients undergoing revision surgery, the decision to perform instrumented posterolateral fusion versus revision discectomy alone was made on the basis of objective radiographic instability criteria applied uniformly throughout the study period, defined as translation exceeding 3 mm or angulation exceeding 10° on dynamic flexion–extension radiographs at the index level, consistent with established lumbar instability thresholds. Segmental translation and angulation were measured on standing dynamic radiographs at the index level, and the corresponding values in patients undergoing fusion are summarized in the Results [20].

### 2.3 Data Collection and Outcome Definition

Demographic and clinical variables extracted from institutional medical records included: age at surgery, sex, smoking status, and the presence of comorbidities, specifically diabetes mellitus (DM) and hypertension (HTN). Smoking status was classified as active smoking at the time of index surgery. Herniation morphology was categorized as extruded or sequestrated based on preoperative MRI interpretation by a senior neurosurgeon. Herniation level, length of hospital stay, intraoperative complications, and early (≤ 30‐day) postoperative adverse events after both the index and revision procedures were also recorded. Body mass index could not be calculated because anthropometric measurements (body weight and height) were not systematically recorded in the institutional dataset during the study period; this is addressed as a study limitation. All other analyzed covariates were completely documented, and no imputation was required.

The primary outcome was reoperation‐confirmed, same‐level ipsilateral recurrent disc herniation, defined as radiologically confirmed reherniation at the same lumbar level and side, associated with recurrent radiculopathy and requiring reoperation, arising after an initial pain‐free interval following the index procedure. This definition excluded technical failure (inadequate primary decompression), residual fragment, and early surgical complications: patients with persistent or immediately recurring radiculopathy within 4 weeks of surgery and therefore without a documented pain‐free interval were classified as technical failures and excluded from the recurrence analysis, irrespective of imaging findings. Patients recorded in the institutional database as having recurrence but lacking both a documented time to recurrence and a confirmed reoperation record were reclassified as non‐events and retained in the analytical cohort (*n* = 2), as they did not satisfy the complete outcome definition for confirmed recurrence. For patients with confirmed recurrence, the following secondary data were collected: time from index surgery to reoperation (months), type of revision procedure (revision discectomy alone or instrumented posterolateral fusion), and the occurrence of any second recurrence during follow‐up. Patients were followed from the date of primary lumbar discectomy until reoperation for ipsilateral same‐level recurrence or the study end date (6 March 2026), whichever occurred first. Scheduled clinical review was performed at the first, sixth, and twelfth postoperative months and annually thereafter. To minimize ascertainment bias arising from recurrences managed outside our institution, institutional follow‐up was supplemented by systematic interrogation of the Turkish national personal health record system (e‐Nabız). Operated by the Republic of Türkiye Ministry of Health and in nationwide use since 2015, e‐Nabız consolidates diagnoses, procedures, imaging studies, laboratory results, and clinical reports generated across public, private, and university healthcare facilities, with data flowing from more than 28,000 integrated health institutions and linked to the national social security reimbursement system [21]. Any lumbar reoperation performed at any licensed healthcare facility in Türkiye generates a corresponding procedural and imaging record within this system. As a result, cross‐institutional surgical events remain identifiable irrespective of where treatment was delivered. Follow‐up status and reoperations performed outside our institution were verified through this system, and no patients were lost to follow‐up.

### 2.4 Statistical Analysis

Continuous variables are presented as mean ± standard deviation (SD) or as median with interquartile range (IQR) where the distribution was skewed. Categorical variables are expressed as counts and percentages. Differences between the recurrence and non‐recurrence groups were evaluated using independent‐samples Student's *t*‐test for normally distributed continuous variables, and the chi‐square test or Fisher's exact test for categorical variables, as appropriate. The corresponding test statistics (*t*‐values and Pearson χ^2^ values) are reported together with the *p*‐values. Time to recurrence was compared between smokers and non‐smokers with the Mann–Whitney *U* test, recurrence rates across herniation levels were compared with the Fisher–Freeman–Halton exact test, and incidence rates were calculated per 1000 person‐years of follow‐up.

Candidate predictors were first examined using univariate binary logistic regression. Given the limited number of events, a screening threshold of *p* < 0.20 was used to select variables for multivariable modeling. This conventional approach reduces overfitting while retaining variables of potential relevance. Comorbidities of established clinical importance (diabetes mellitus and hypertension) were retained on both statistical and clinical grounds. Multivariable binary logistic regression was performed using maximum likelihood estimation. Because independent‐predictor inference is constrained by the limited number of events, model selection was guided by the Akaike Information Criterion (AIC): candidate models were compared and the most parsimonious model with the lowest AIC was retained as the primary model. The final primary model comprised diabetes mellitus and hypertension, yielding an events‐per‐variable (EPV) ratio of 14.0, above the conventional threshold of 10. A fully adjusted model additionally including age and sex was fitted and is reported alongside the primary model, ensuring that clinically important covariates were not excluded solely on the basis of univariate statistical thresholds. The two models yielded concordant estimates for diabetes mellitus and hypertension, indicating that the primary findings are not an artifact of the variable‐selection strategy. To further address the sparse‐data setting and to provide confidence intervals and *p*‐values that remain robust under small event counts, Firth penalized logistic regression was also performed [22]. The maximum‐likelihood and Firth‐penalized models yielded similar effect estimates (hypertension OR 3.42 vs. 3.54; diabetes OR 3.17 vs. 3.29), supporting model stability; full Firth confidence intervals and *p*‐values are reported with the primary‐model estimates. Multicollinearity was assessed using variance inflation factors (VIF); all values were below 2.0, indicating no meaningful collinearity. Age met the *p* < 0.20 screening threshold (*p* = 0.136) and was therefore evaluated in multivariable modeling; however, its inclusion increased the AIC and it did not contribute independently (adjusted OR 1.01, *p* = 0.351). Although age did not improve model fit, it is reported because of its established clinical relevance. Sex did not meet the screening threshold (*p* = 0.848) and was not entered; both age and sex were nonetheless examined in the fully adjusted model without materially altering the estimates for diabetes mellitus and hypertension.

The Kaplan–Meier method was used to estimate recurrence‐free survival, and the log‐rank test (reported with its χ^2^ statistic) was used to evaluate between‐group differences. Multivariable Cox proportional hazards regression was performed to estimate adjusted hazard ratios (HRs) with 95% confidence intervals (CIs). The proportional hazards assumption was formally tested using scaled Schoenfeld residuals, with no significant violation detected for any included covariate (all *p* > 0.05). Model discrimination was quantified using Harrell's concordance index (C‐index). The chi‐square test or Fisher's exact test was used for all categorical comparisons within the Results section. Descriptive statistics, group comparisons, Kaplan–Meier survival analysis, and the log‐rank test were performed using IBM SPSS Statistics (version 28.0; IBM Corp., Armonk, NY, USA). Multivariable binary logistic regression, Firth penalized logistic regression, and Cox proportional hazards modeling were performed in Python (version 3.11) using the statsmodels and lifelines libraries; Firth penalized estimation was implemented through penalized maximum‐likelihood estimation, and proportional‐hazards diagnostics were obtained with lifelines. All results were cross‐checked by the authors against the source dataset. A two‐sided *p*‐value < 0.05 was considered statistically significant.

## 3 Results

### 3.1 Patient Characteristics

A total of 432 patients who underwent primary lumbar discectomy during the study period satisfied all inclusion and exclusion criteria. Two patients initially recorded as recurrence in the institutional database lacked both a documented time‐to‐recurrence and a confirmed reoperation record; these patients were reclassified as non‐events and retained in the analytical cohort, resulting in an analytical cohort of 432 patients, including 28 confirmed recurrence events and 404 non‐recurrence observations. The mean age at surgery was 46.5 ± 12.5 years (range 18–81 years), and 223 patients (51.6%) were male. Median follow‐up duration for the entire cohort was 85 months (IQR 50–103 months). Active smoking at the time of index surgery was documented in 164 patients (38.0%). Diabetes mellitus was present in 23 patients (5.3%) and hypertension in 22 (5.1%). Regarding herniation morphology, sequestrated herniation was identified in 212 patients (49.1%) and extruded herniation in 220 (50.9%). The most commonly affected levels were L4–5 (*n* = 229, 53.0%) and L5–S1 (*n* = 159, 36.8%), followed by L3–4 (*n* = 25, 5.8%), L2–3 (*n* = 16, 3.7%), and L1–2 (*n* = 3, 0.7%). The median length of hospital stay was 5 days (IQR 4–5 days). Baseline characteristics did not differ significantly between the recurrence and non‐recurrence groups with respect to age (*t* = 1.49, *p* = 0.136), sex (χ^2^ = 0.05, *p* = 0.848), active smoking (χ^2^ = 1.12, *p* = 0.321), or herniation morphology (χ^2^ = 1.62, *p* = 0.243), whereas diabetes mellitus (χ^2^ = 9.33, *p* = 0.012) and hypertension (χ^2^ = 10.09, *p* = 0.010) were more frequent in the recurrence group (Table 1).

**TABLE 1:** Baseline demographic and clinical characteristics of the study cohort (*n* = 432).

Variable	Overall (*n* = 432)	Recurrence (*n* = 28)	No Recurrence (*n* = 404)	Test statistic, *p*‐value
Demographics				
Age at surgery, mean ± SD (years)	46.5 ± 12.5	49.9 ± 10.5	46.2 ± 12.6	t = 1.49, *p* = 0.136
Male sex, *n* (%)	223 (51.6)	15 (53.6)	208 (51.5)	χ^2^ = 0.05, *p* = 0.848
Comorbidities				
Active smoking, *n* (%)	164 (38.0)	8 (28.6)	156 (38.6)	χ^2^ = 1.12, *p* = 0.321
Diabetes mellitus, *n* (%)	23 (5.3)	5 (17.9)	18 (4.5)	χ^2^ = 9.33, *p* = 0.012
Hypertension, *n* (%)	22 (5.1)	5 (17.9)	17 (4.2)	χ^2^ = 10.09, *p* = 0.010
Herniation characteristics				
Sequestrated morphology, *n* (%)	212 (49.1)	17 (60.7)	195 (48.3)	χ^2^ = 1.62, *p* = 0.243
Herniation level *n* (%)				χ^2^ = 0.57, *p* = 0.98
L4–5	229 (53.0)	16 (57.1)	213 (52.7)	
L5–S1	159 (36.8)	10 (35.7)	149 (36.9)	
L3–4	25 (5.8)	1 (3.6)	24 (5.9)	
L2–3	16 (3.7)	1 (3.6)	15 (3.7)	
L1–2	3 (0.7)	0 (0.0)	3 (0.7)	
Perioperation				
CSF leak (incidental durotomy), *n* (%)	15 (3.5)	0 (0.0)	15 (3.7)	
Length of hospital stay, median (IQR), days	5 (4–5)	5 (4–6)	5 (4–5)	
Follow‐up				
Follow‐up duration, median (IQR), months†	85 (50–103)	18.5 (10–36)	89 (57–104)	

*Note:* Continuous variables are presented as mean ± SD or median (IQR); categorical variables as *n* (%). Comparisons by independent‐samples *t*‐test (age; *t* statistic shown) or Fisher's exact test (categorical variables; Pearson χ^2^ statistic shown with the Fisher's exact *p*‐value; Fisher–Freeman–Halton exact test for herniation level). Blank cells indicate that no significance test was performed.

Abbreviations: CSF = cerebrospinal fluid; DM = diabetes mellitus; HTN = hypertension; IQR = interquartile range; SD = standard deviation.

^†^ Follow‐up in the recurrence group reflects time to the recurrence event and is shown descriptively; no significance test is reported for this row.

A sensitivity analysis confirmed that the classification of these two cases did not affect the findings: reclassifying both as recurrences (increasing the event count to 30; recurrence rate 6.9%) or excluding them entirely (*n* = 430) left the recurrence rate and the associations of diabetes mellitus and hypertension unchanged in direction, magnitude, and statistical significance across logistic, Cox, and log‐rank analyses.

### 3.2 Recurrence Rate and Timing

Recurrence requiring surgical intervention was identified in 28 of 432 patients, corresponding to an overall reoperation‐confirmed recurrence rate of 6.5%. The mean time from index surgery to recurrence was 23.7 ± 19.5 months (median 18.5 months; range 1–73 months). The temporal distribution was predominantly late: 19 patients (67.9%) experienced recurrence at 12 months or beyond the index procedure, while 9 patients (32.1%) presented within the first postoperative year. The largest share of recurrences (*n* = 13, 46.4%) occurred between 12 and 36 months. The 164 active smokers (38.0%) contributed 967.9 person‐years of follow‐up, and the 268 non‐smokers contributed 1816.2 person‐years. Recurrence occurred in 8 smokers (4.9%; 8.3 per 1000 person‐years) and in 20 of 268 non‐smokers (7.5%; 11.0 per 1000 person‐years). Among the 8 smokers with recurrence, the mean time to recurrence was 22.0 ± 21.4 months (median 16.5 months; IQR 2.5–43.0; range 1–52 months), and 4 of these 8 events (50.0%) occurred within the first postoperative year. Among the 20 non‐smokers with recurrence, the mean time to recurrence was 24.4 ± 19.3 months (median 18.5 months; IQR 13.0–28.5; range 2–73 months), with 5 events (25.0%) within the first year. The time to recurrence did not differ significantly between smokers and non‐smokers (Mann–Whitney U = 68.5, *p* = 0.575), nor did the proportion of recurrences within the first year (Fisher's exact test, *p* = 0.371).

Recurrence rates did not differ significantly across herniation levels: 7.0% at L4–5 (16/229), 6.3% at L5–S1 (10/159), 4.0% at L3–4 (1/25), 6.3% at L2–3 (1/16), and 0% at L1–2 (0/3) (χ^2^ = 0.57; Fisher–Freeman–Halton exact test, *p* = 0.98). Similarly, herniation morphology (extruded vs. sequestrated) was not significantly associated with recurrence (χ^2^ = 1.62, *p* = 0.243).

### 3.3 Surgical Management of Recurrence

Among the 28 patients who developed recurrence, the surgical strategy at reoperation was determined by dynamic flexion–extension radiographic evaluation of segmental stability, applying predefined instability thresholds uniformly across the study period. Instrumented posterolateral fusion was performed in 21 patients (75.0%) who demonstrated objective radiographic instability. In these 21 patients, the mean segmental translation was 4.2 ± 1.1 mm and the mean angulation was 12.2° ± 2.1° on dynamic flexion–extension radiographs, both exceeding the predefined thresholds of 3 mm and 10°. The remaining 7 patients (25.0%), who did not meet these instability criteria, were managed with revision discectomy alone. No patient experienced a second recurrence during a median post‐revision follow‐up of 61.5 months (IQR 35–89 months), irrespective of whether fusion or revision discectomy alone was performed.

### 3.4 Perioperative Complications and Early Adverse Events

Intraoperative complications during the index procedure were limited to incidental durotomy with cerebrospinal fluid leakage, which occurred in 15 patients (3.5%). All durotomies were recognized intraoperatively by visible cerebrospinal fluid egress from the dural tear and were managed with primary dural repair during the same procedure. None of these patients required reoperation for cerebrospinal fluid leakage or developed a symptomatic pseudomeningocele, and all recovered without sequelae; their median length of hospital stay was 12 days (IQR 10–14 days). No other intraoperative complications and no other adverse events within 30 days of the index procedure, including surgical site infection, hematoma, new neurological deficit, thromboembolic events, readmission, or death, were documented. No intraoperative complications occurred during the 28 revision procedures. Within 30 days of revision surgery, 2 patients (7.1%) developed a surgical site infection, one after instrumented fusion and one after revision discectomy alone, and both infections presented within the first two postoperative weeks. The infection after fusion required readmission, surgical debridement, and intravenous antibiotic therapy, and the instrumentation was retained. The infection after revision discectomy alone was treated with antibiotic therapy without surgical intervention. Both infections resolved completely without sequelae. No other adverse events, including hematoma, new neurological deficit, thromboembolic events, or death, occurred within 30 days of revision surgery.

### 3.5 Risk Factor Analysis

*Univariate analysis*. In univariate comparison between the recurrence (*n* = 28) and non‐recurrence (*n* = 404) groups, hypertension (OR 4.95, 95% CI 1.68–14.61; *p* = 0.010) and diabetes mellitus (OR 4.66, 95% CI 1.59–13.68; *p* = 0.012) were significantly associated with recurrence. Older age showed a non‐significant trend (49.9 ± 10.5 vs. 46.2 ± 12.6 years; t = 1.49, *p* = 0.136), and sequestrated morphology was more frequent in the recurrence group without reaching significance (OR 1.66, 95% CI 0.76–3.62; *p* = 0.243). Active smoking was not associated with recurrence (OR 0.64, 95% CI 0.27–1.48; *p* = 0.321), nor was male sex (OR 1.09, 95% CI 0.50–2.34; *p* = 0.848) (Table 2).

**TABLE 2:** Univariate and multivariable binary logistic regression for factors associated with reoperation‐confirmed recurrence.

Part A. Primary model (diabetes mellitus + hypertension)			
Variable	Univariate OR (95% CI), *p*	Multivariable OR (95% CI), *p*	Firth OR (95% CI), *p*
Hypertension	4.95 (1.68–14.61), 0.010	3.42 (1.04–11.21), 0.043	3.54 (1.10–11.36), 0.033
Diabetes mellitus	4.66 (1.59–13.68), 0.012	3.17 (0.97–10.35), 0.056	3.29 (1.03–10.51), 0.044

Part B. Fully adjusted model (diabetes mellitus + hypertension + age + sex)		
Variable	Adjusted OR (95% CI)	*p*‐value
Hypertension	3.27 (0.99–10.79)	0.052
Diabetes mellitus	2.84 (0.85–9.53)	0.091
Age (per year)	1.01 (0.98–1.05)	0.351
Male sex	1.03 (0.47–2.27)	0.932

*Note:* Firth penalized logistic regression estimates are reported with full confidence intervals and *p*‐values. Primary model fit: AIC = 203.6; McFadden R^2^ = 0.047; likelihood‐ratio test *p* = 0.008; EPV = 14.0; VIF < 2.0 for all predictors. Variables screened but not retained (univariate): age (*p* = 0.136), active smoking (OR 0.64, 95% CI 0.27–1.48; *p* = 0.321), sequestrated morphology (OR 1.66, 95% CI 0.76–3.62; *p* = 0.243) and male sex (OR 1.09, 95% CI 0.50–2.34; *p* = 0.848); smoking was not associated with recurrence.

Abbreviations: CI, confidence interval; EPV, events per variable; OR, odds ratio; VIF, variance inflation factor.

*Multivariable analysis*. In the primary maximum‐likelihood logistic model comprising diabetes mellitus and hypertension, hypertension was associated with recurrence (adjusted OR 3.42, 95% CI 1.04–11.21; *p* = 0.043) and diabetes mellitus showed an association of similar magnitude that did not reach significance under maximum‐likelihood estimation (adjusted OR 3.17, 95% CI 0.97–10.35; *p* = 0.056) (model AIC 203.6; McFadden R^2^ 0.047; likelihood‐ratio *p* = 0.008; EPV 14.0). In the fully adjusted model additionally including age and sex, the point estimates were essentially unchanged (hypertension OR 3.27, 95% CI 0.99–10.79, *p* = 0.052; diabetes OR 2.84, 95% CI 0.85–9.53, *p* = 0.091), with neither age (OR 1.01, *p* = 0.351) nor sex (OR 1.03, *p* = 0.932) contributing independently. In the secondary Firth penalized logistic regression, the estimates were concordant and both comorbidities reached significance (hypertension OR 3.54, 95% CI 1.10–11.36, *p* = 0.033; diabetes OR 3.29, 95% CI 1.03–10.51, *p* = 0.044) (Table 2).

*Survival analysis*. Kaplan–Meier analysis demonstrated recurrence‐free survival rates of 97.9%, 95.8%, 94.8%, 93.7%, and 93.0% at 1, 2, 3, 5, and 10 years, respectively, for the overall cohort; the 10‐year estimate should be interpreted cautiously, as only 40 patients remained at risk at that time point (Figure 1). Log‐rank testing showed significantly lower recurrence‐free survival among patients with diabetes mellitus (5‐year 82.4% vs. 94.3%; χ^2^ = 10.16, *p* = 0.001) and hypertension (5‐year 80.6% vs. 94.4%; χ^2^ = 10.75, *p* = 0.001), whereas recurrence‐free survival did not differ by smoking status (5‐year 94.6% in smokers vs. 93.1% in non‐smokers; χ^2^ = 0.91, *p* = 0.340) (Figure 2; Table 3). In multivariable Cox proportional hazards analysis, both comorbidities were associated with earlier recurrence in the primary model (hypertension HR 3.27, 95% CI 1.16–9.17, *p* = 0.024; diabetes HR 3.14, 95% CI 1.12–8.80, *p* = 0.030; C‐index 0.565), and estimates were maintained in the fully adjusted model (hypertension HR 3.18, *p* = 0.028; diabetes HR 2.84, *p* = 0.051; C‐index 0.611). Proportional hazards assumptions were satisfied for all covariates (scaled Schoenfeld residuals, all *p* > 0.05). Hypertension was associated with recurrence in the primary maximum‐likelihood logistic model, in Firth penalized logistic regression, in the primary Cox model, and in the log‐rank comparison, whereas in the fully adjusted logistic model it fell just short of significance (*p* = 0.052). Diabetes mellitus was associated with recurrence in the Firth penalized, Cox, and log‐rank analyses but not in the primary maximum‐likelihood logistic model (*p* = 0.056). Smoking was not associated with recurrence in any analysis. The adjusted effect estimates are summarized in Figure 3.

**FIGURE 1:**
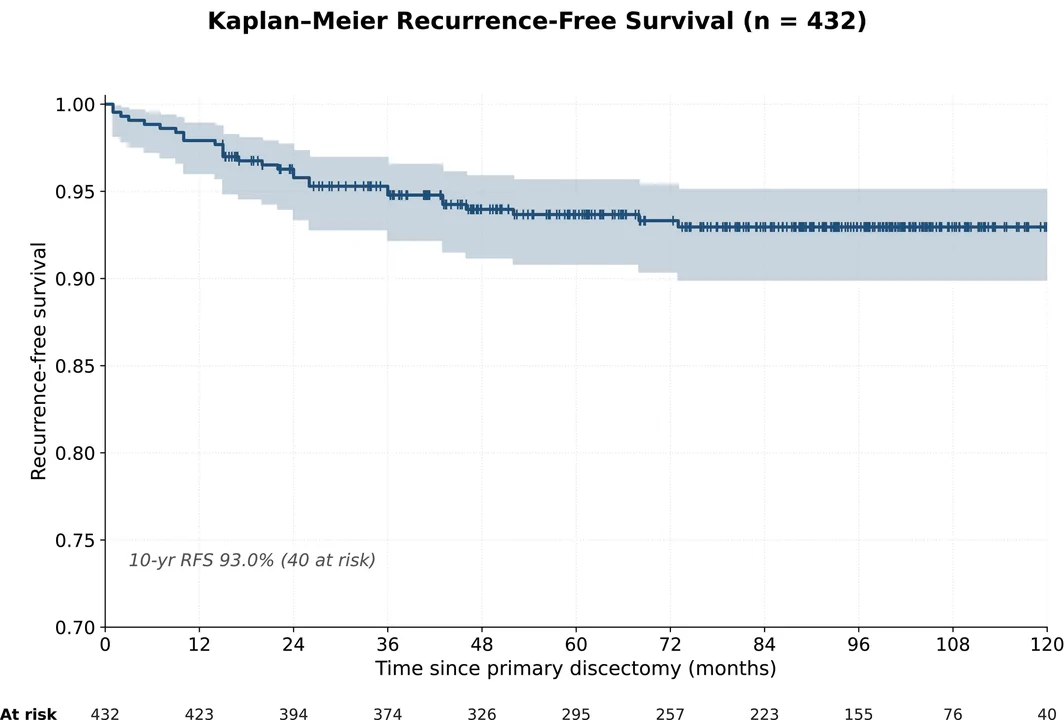
Kaplan–Meier recurrence‐free survival curve for the overall cohort (*n* = 432). Recurrence‐free survival was 97.9% at 1 year, 95.8% at 2 years, 94.8% at 3 years, 93.7% at 5 years, and 93.0% at 10 years. The shaded band represents the 95% confidence interval (complementary log–log method). Vertical tick marks indicate censored observations, and the number of patients at risk is shown below the time axis. The 10‐year estimate should be interpreted with caution, as only 40 patients remained at risk at that time point.

**FIGURE 2:**
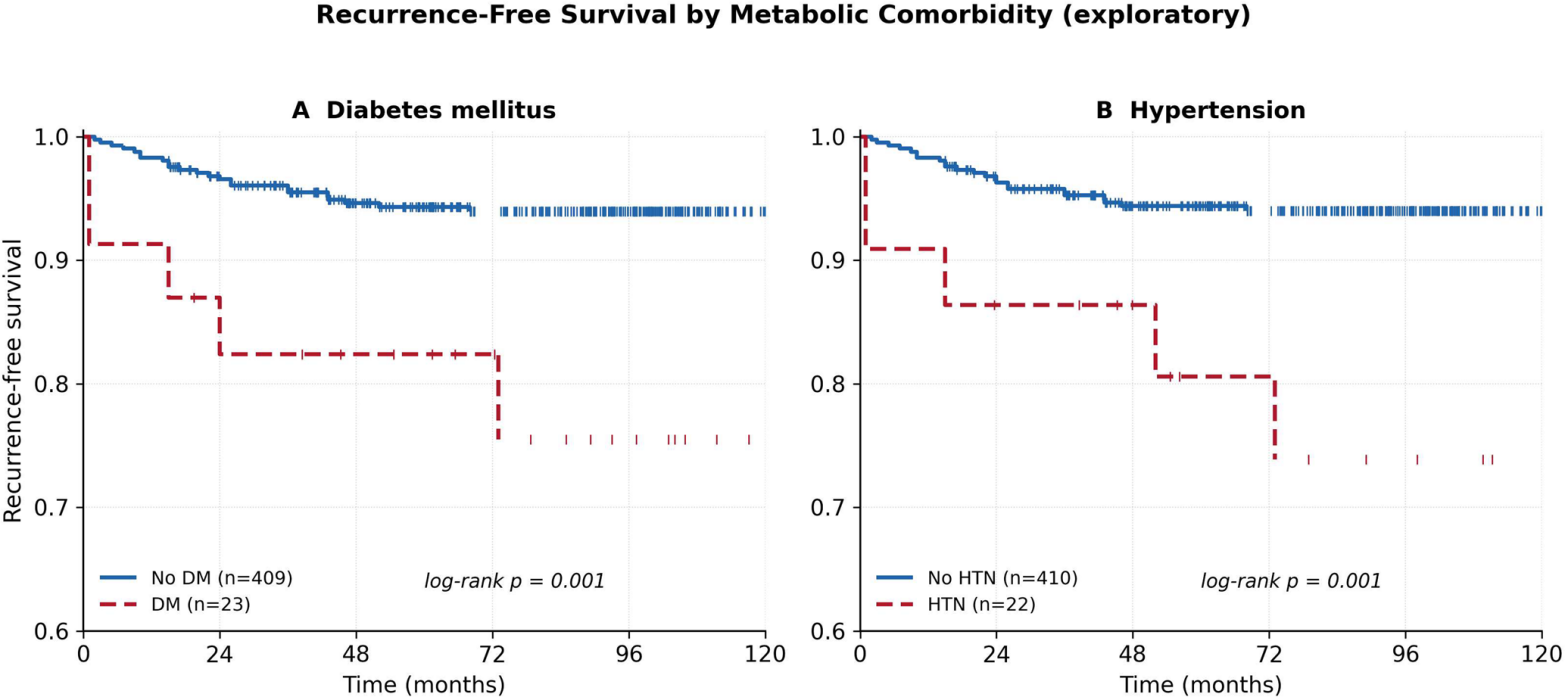
Kaplan–Meier recurrence‐free survival stratified by metabolic comorbidity (exploratory analysis). A: Diabetes mellitus subgroups (no DM, *n* = 409 vs. DM, *n* = 23; log‐rank χ^2^ = 10.16, *p* = 0.001); 5‐year recurrence‐free survival 94.3% versus 82.4%. B: Hypertension subgroups (no HTN, *n* = 410 vs. HTN, *n* = 22; log‐rank χ^2^ = 10.75, *p* = 0.001); 5‐year recurrence‐free survival 94.4% versus 80.6%. Tick marks indicate censored observations. These analyses are based on only five recurrence events in each comorbidity subgroup and are therefore severely underpowered; the curves are presented for hypothesis‐generating purposes only and should not be interpreted as confirmatory evidence of an independent association.

**TABLE 3:** Multivariable Cox proportional hazards regression and Kaplan–Meier recurrence‐free survival estimates.

Part A. Cox proportional hazards regression		
Model/Variable	Adjusted HR (95% CI)	*p*‐value
Primary model (DM + HTN)		
Hypertension	3.27 (1.16–9.17)	0.024
Diabetes mellitus	3.14 (1.12–8.80)	0.030
Fully adjusted model (DM + HTN + age + sex)		
Hypertension	3.18 (1.13–8.90)	0.028
Diabetes mellitus	2.84 (0.99–8.10)	0.051
Age (per year)	1.02 (0.99–1.04)	0.302
Male sex	1.05 (0.50–2.22)	0.894

Part B. Kaplan–Meier recurrence‐free survival (%) at key time points					
Time point	Overall cohort	No DM	DM (+)	No HTN	HTN (+)
1 year	97.9	98.3	91.3	98.3	90.9
2 years	95.8	96.5	82.4	96.3	86.4
5 years	93.7	94.3	82.4	94.4	80.6
10 years	93.0	93.9	75.5	94.0	73.9

*Note:* C‐index 0.565 (primary model) and 0.611 (fully adjusted model); scaled Schoenfeld residual test: all *p > 0.05* (proportional hazards assumption satisfied). Log‐rank test: DM χ^2^ = 10.16, *p* = 0.001; HTN χ^2^ = 10.75, *p* = 0.001. DM (+) and HTN (+) subgroups contained only 5 recurrence events each; the corresponding estimates are underpowered and hypothesis‐generating. The 10‐year estimates should be interpreted with particular caution, as only 40 patients remained at risk in the overall cohort at that time point.

**FIGURE 3:**
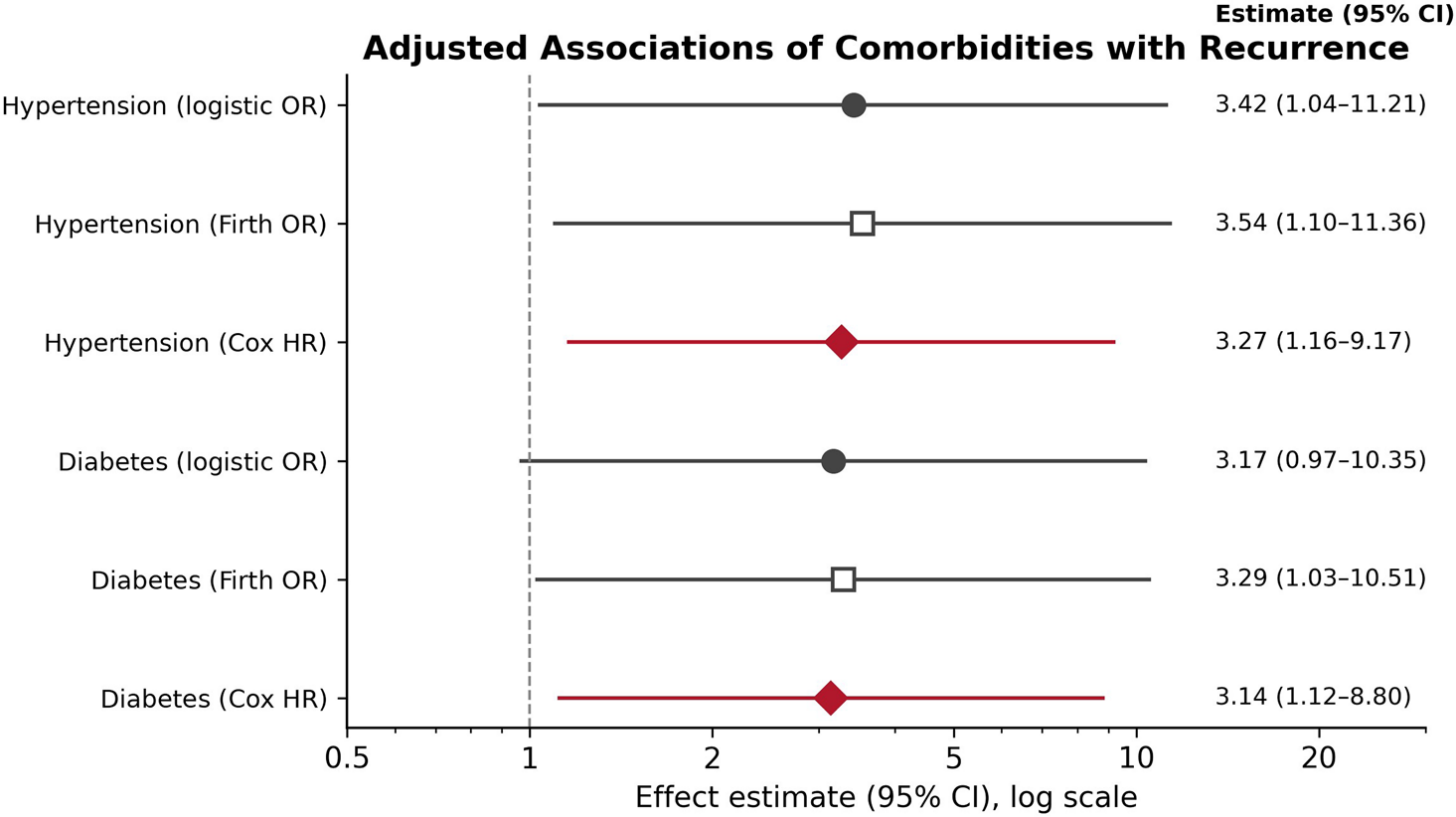
Forest plot of adjusted effect estimates for the association of diabetes mellitus and hypertension with reoperation‐confirmed recurrence. For each comorbidity, three adjusted estimates from the primary models are shown: the odds ratio (OR) from maximum‐likelihood logistic regression (filled circle), the Firth penalized OR (open square), and the hazard ratio (HR) from Cox proportional hazards regression (diamond). Horizontal lines denote 95% confidence intervals, and the dashed vertical line marks the null value (1.0). Estimates are displayed on a logarithmic scale. Both comorbidities showed positive associations in all three approaches, although the maximum‐likelihood estimate for diabetes mellitus did not reach statistical significance; given the limited number of events, these associations are hypothesis‐generating rather than confirmatory.

## 4 Discussion

In this retrospective cohort of 432 patients who underwent primary lumbar discectomy over a 10‐year period, reoperation‐confirmed recurrence occurred in 6.5% of patients and followed a predominantly late temporal course, with approximately two thirds of events arising beyond the first postoperative year (median 18.5 months). At revision, instrumented posterolateral fusion was required in 75% of cases for objectively documented segmental instability, and no second recurrence was observed. Hypertension was associated with recurrence in the primary logistic model and in the Firth penalized, Cox, and log‐rank analyses, whereas the association of diabetes mellitus did not reach significance in the primary logistic model and was supported only by secondary analyses; active smoking was not associated with recurrence. The main contribution of this study is that it describes the recurrence rate, its temporal distribution, the surgical profile at revision, and the associated comorbidities together, in a single cohort with complete nationwide follow‐up. These parameters are rarely reported together and are relevant to postoperative surveillance and revision planning.

The observed recurrence rate of 6.5% aligns with the mid‐range of the established literature, in which reoperation‐confirmed recurrence rates of 5%–11% are reported [13, 14, 15]. The 2024 World Federation of Neurosurgical Societies Spine Committee systematic review documented a pooled recurrence rate of approximately 5% [14], while a recent nationwide cohort reported a 5‐year reoperation rate of 14.4% [11]. Our estimate is consistent with these figures and reflects a stringent, reoperation‐confirmed, same‐level endpoint that excludes technical failure, residual fragment, and early surgical complications. As with any reoperation‐based definition, this conservative approach necessarily underestimates the true biological recurrence rate, because symptomatic recurrences managed nonoperatively are not captured; however, it ensures that every counted event represents a clinically significant outcome requiring intervention.

The temporal distribution of recurrence in our cohort was predominantly late, with a mean time to reoperation of 23.7 ± 19.5 months (median 18.5 months) and 68% of events occurring beyond the first postoperative year. This timing lies toward the later end of the reported spectrum. In a nationwide Korean cohort, half of all reoperations occurred within the first postoperative year [12], and Matsumoto et al. reported a mean time to recurrence of 16.6 months after microendoscopic discectomy, with 54% of recurrences within the first year, compared with only 32% within the first year in our cohort [23]. The predominantly late pattern in our series may reflect the consistent use of a standardized microsurgical technique with limited intradiscal curettage, which preserves disc height and segmental stability, together with a predominantly working‐age population in whom continued mechanical loading may drive gradual degeneration of a structurally weakened annulus. Regardless of the mechanism, this late distribution has a clinical implication: short‐term (12‐month) surveillance is insufficient to capture the true burden of recurrence, and longer follow‐up is needed.

Among the 28 patients with recurrence, instrumented posterolateral fusion was performed in 21 (75%), a proportion substantially higher than the 43.4% reported in a nationwide United States analysis of recurrent disc herniation [24]. This difference is likely multifactorial and is best interpreted in light of the protracted time to recurrence in our cohort: the longer interval to reoperation allows greater disc‐height loss and progressive segmental degeneration, increasing the likelihood that objective instability criteria are met at revision. A recent meta‐analysis, a systematic review, and a prospective comparative study support instrumented fusion in the specific setting of demonstrable segmental instability [25, 26, 27]. However, the high fusion proportion should be interpreted with caution. Instability was assessed on dynamic radiographs in all revision patients, and in the 21 fused patients the mean translation and angulation (4.2 ± 1.1 mm and 12.2° ± 2.1°) exceeded the predefined thresholds. However, disc‐height loss and the interobserver reliability of these measurements were not assessed, so the indication for fusion rests on the applied radiographic thresholds alone. The complete absence of a second recurrence (0/28) is clinically reassuring, but with only 28 reoperated patients, 21 of whom underwent fusion, and no adjusted comparison between revision strategies, these data cannot establish that fusion is superior to revision discectomy alone or that it independently prevents further recurrence. This finding is therefore best regarded as a descriptive observation rather than as evidence of comparative efficacy, and it remains subject to indication bias.

Diabetes mellitus and hypertension were each associated with recurrence in univariate analysis, and both associations pointed in the same direction in the maximum‐likelihood multivariable, Firth penalized, and Cox proportional hazards analyses and in the log‐rank comparisons of recurrence‐free survival. Statistical significance, however, differed between the two comorbidities. Hypertension remained significant in the primary maximum‐likelihood model, whereas diabetes mellitus did not (adjusted OR 3.17, 95% CI 0.97–10.35; *p* = 0.056) and reached significance only in the secondary Firth penalized and time‐to‐event analyses. The evidence for diabetes mellitus should therefore be regarded as weaker than that for hypertension. Mechanistically, both conditions can compromise the microvascular supply of the avascular intervertebral disc and impair annular healing after discectomy, providing biological plausibility for a link with recurrence [17, 18, 28]. These observations should still be framed as associations rather than as independent or causal predictors: each comorbidity subgroup contained only five recurrence events, the confidence intervals are wide, and residual confounding, most notably by unmeasured body mass index, cannot be excluded. We therefore present hypertension, and to a lesser extent diabetes mellitus, as hypothesis‐generating correlates of recurrence that merit confirmation in adequately powered multicenter cohorts. Accordingly, we do not describe either comorbidity as an independent predictor of recurrence.

In our data, active smoking was not associated with recurrence (OR 0.64, 95% CI 0.27–1.48; *p* = 0.321; log‐rank *p* = 0.340). Earlier meta‐analyses identified smoking as a patient‐related risk factor, with pooled estimates indicating up to a doubling of recurrence risk [16, 29]. Two recent large meta‐analyses, however, report a smaller or nonsignificant effect, and our estimate is best interpreted alongside them. In a 2026 meta‐analysis of 52,479 patients from 51 studies, Soliman et al. found that smoking was not a significant predictor of recurrent disc herniation (OR 1.16, 95% CI 1.00–1.33). The only significant predictors in that meta‐analysis were high body mass index, diabetes (OR 1.48, 95% CI 1.23–1.77), increased sagittal range of motion, and Modic changes [30]. In a meta‐analysis of 1,031,348 patients that focused on reoperation, Samir et al. estimated a modest smoking effect (OR 1.39, 95% CI 1.09–1.78) [31]. Our confidence interval (0.27–1.48) includes the null value and overlaps the lower part of the interval reported by Samir et al. Our null finding is therefore concordant with the absence of a significant smoking effect in the meta‐analysis by Soliman et al., and it does not contradict the modest effect reported by Samir et al., given the limited power of a cohort with 28 events. Similarly, our diabetes estimate is directionally concordant with the pooled diabetes effect reported by Soliman et al. In contrast, hypertension was not among the significant predictors identified in that meta‐analysis, which further supports regarding our hypertension finding as hypothesis‐generating. In our cohort, recurrence occurred at 8.3 per 1000 person‐years among smokers and 11.0 per 1000 person‐years among non‐smokers, and the time to recurrence among smokers (median 16.5 months; range 1–52 months) did not differ significantly from that among non‐smokers (median 18.5 months; *p* = 0.575). Several factors may explain why our point estimate lies below 1.0, in contrast to earlier meta‐analyses, and caution against overinterpreting a single‐center null result. Smoking was recorded only as active status at the time of index surgery, without pack‐year history or biochemical verification, so exposure misclassification is likely and precludes any dose–response assessment. In addition, the limited number of events constrains statistical power. Our finding should therefore be read as an absence of a detectable association in this cohort rather than as evidence that smoking is unrelated to recurrence; the established experimental and epidemiological literature linking nicotine to impaired disc nutrition and annular healing remains relevant [32, 33].

These findings carry several clinical implications. First, because recurrence was predominantly late (median 18.5 months), postoperative surveillance should extend well beyond the conventional 12‐month window; follow‐up through at least three to five years appears prudent, particularly in patients with metabolic or vascular comorbidity. Second, the high revision‐fusion proportion and the absence of second recurrence support the use of a standardized instability assessment, using dynamic flexion–extension radiography as a minimum, when planning revision surgery, so that fusion is reserved for objectively unstable segments. Third, the associations of hypertension and, less robustly, diabetes mellitus with recurrence suggest that these common comorbidities may deserve attention in preoperative counseling and risk communication; however, given the limited number of events, our data do not support a formal, quantitatively weighted risk‐stratification score, and any such tool would require external multicenter derivation and validation before clinical use. We deliberately refrain from proposing smoking‐based counseling as a study‐specific recommendation, as smoking was not associated with recurrence in this cohort.

This study has several strengths. Few contemporary cohorts have reported recurrence timing, revision strategy, and long‐term post‐revision outcomes in the same patients. The 10‐year consecutive design is among the longest follow‐up periods in the recurrent lumbar disc herniation literature and captured late recurrences that shorter studies often miss. Follow‐up completeness was further reinforced by systematic interrogation of the national electronic health record system, which enabled nationwide ascertainment of reoperations irrespective of the treating institution. This ensures complete ascertainment of the primary endpoint (reoperation‐confirmed recurrence) rather than complete longitudinal clinical follow‐up: patients without a recorded reoperation were confirmed free of the primary endpoint through the study end date, although the detail of their interim clinical follow‐up varied. Finally, the use of four complementary analytical approaches allowed us to examine the robustness of the comorbidity associations.

Several limitations must be acknowledged. First, the retrospective single‐center design limits external generalizability; surgical indications, reoperation thresholds, and fusion decision‐making may reflect local practice, although consecutive recruitment and a uniform surgical technique mitigate this to some degree. Second, the reoperation‐confirmed endpoint underestimates the true biological recurrence rate. Third, although the primary model achieved an events‐per‐variable ratio of 14.0, only 28 recurrence events were observed overall, so the comorbidity subgroup analyses remain underpowered and yield wide confidence intervals. Fourth, body mass index could not be calculated because anthropometric measurements were not systematically recorded, and several potentially relevant variables were unavailable, including occupational physical load, annular defect size, disc degeneration grade, Modic changes, disc height, facet degeneration, and postoperative rehabilitation status; their absence may bias the observed comorbidity associations and limits causal interpretation. Fifth, quantitative translation and angulation values are reported only for the fused patients, and disc‐height loss and the interobserver reliability of the instability measurements were not assessed. Sixth, no validated patient‐reported outcome measures (such as the Oswestry Disability Index, visual analog scale, Japanese Orthopedic Association score, EQ‐5D, or return‐to‐work data) were available, precluding assessment of functional recovery, which is the ultimate goal of both primary and revision surgery. Seventh, the 10‐year recurrence‐free survival estimate should be interpreted cautiously, as only 40 patients remained at risk at that time point. Finally, the cohort was treated exclusively with open posterior microsurgical discectomy; because endoscopic and minimally invasive approaches report recurrence rates of 3%–8% in contemporary series, the present findings may not be directly generalizable to these techniques.

These limitations define a clear agenda for future work. Prospective, multicenter studies with standardized recurrence definitions, uniform instability criteria, systematic collection of anthropometric and metabolic data, and validated patient‐reported outcome measures are needed to confirm the comorbidity associations observed here, to clarify the role of smoking with quantitative exposure metrics, and to compare revision discectomy with instrumented fusion under adjusted, indication‐controlled conditions. Multicenter validation would also establish whether the late temporal pattern and the surveillance recommendations derived from this cohort generalize across differing practice settings.

## 5 Conclusion

In this 10‐year single‐center cohort of 432 patients, reoperation‐confirmed recurrence after primary lumbar discectomy occurred in 6.5% and was predominantly late, with approximately two thirds of events arising beyond the first postoperative year (median 18.5 months). Three quarters of revision procedures required instrumented fusion for objectively documented segmental instability, and no second recurrence was observed. Hypertension was associated with recurrence in the primary logistic model and in the Firth penalized, Cox, and log‐rank analyses. Diabetes mellitus showed an association of similar magnitude that did not reach significance in the primary logistic model and was supported only by secondary analyses. Active smoking was not associated with recurrence. Given the limited number of events, these associations are hypothesis‐generating rather than confirmatory. Overall, these findings support postoperative surveillance extending well beyond the first year and instability‐guided decision‐making at revision, and they identify hypertension and diabetes mellitus as candidate correlates that require confirmation in adequately powered multicenter studies.

## Author Contributions

**Serhat Yıldızhan:** software. **Usame Rakip:** data curation. **İhsan Canbek:** visualization. **Mehmet Gazi Boyacı:** writing – original draft. **Serhat Korkmaz:** conceptualization. **Adem Aslan:** writing – review and editing.

## Funding

This research received no specific grant from any funding agency in the public, commercial, or not‐for‐profit sectors.

## Disclosure

This study was reported in accordance with the Strengthening the Reporting of Observational Studies in Epidemiology (STROBE) guidelines for cohort studies. Clinical trial number: not applicable. During the preparation and revision of this manuscript, the authors used AI‐based tools (Claude, Anthropic; and the Springer Nature AI writing assistant) to assist with language editing, drafting and restructuring of manuscript text, formatting of tables, preparation of figures and the graphical abstract, statistical code preparation, computational support, and presentation of statistical output. All statistical analyses were performed using the statistical software specified in the Methods (IBM SPSS Statistics and Python with the statsmodels and lifelines libraries), and all results were independently cross‐checked and verified by the authors against the source dataset. All primary data were extracted from institutional records and the national electronic health record system by the authors. All clinical interpretation, scientific reasoning and conclusions are those of the authors, who reviewed and edited all AI‐assisted output and accept full responsibility for the content of this publication.

## Ethics Statement

This study was approved by the Institutional Ethics Committee of Afyonkarahisar Health Sciences University (meeting no. 2026/3, decision no. 208, dated 06.03.2026). All procedures were conducted in accordance with the Declaration of Helsinki.

## Consent

The informed consent requirement was waived by the Institutional Ethics Committee due to the retrospective nature of the study and the use of de‐identified administrative data.

## Conflicts of Interest

The authors declare no conflicts of interest.

## Data Availability

The datasets generated and/or analyzed during the current study are available from the corresponding author on reasonable request.
